# Mapping Gene Markers for Apple Fruit Ring Rot Disease Resistance Using a Multi-omics Approach

**DOI:** 10.1534/g3.119.400167

**Published:** 2019-03-25

**Authors:** Fei Shen, Zhenyu Huang, Baoguo Zhang, Yi Wang, Xi Zhang, Ting Wu, Xuefeng Xu, Xinzhong Zhang, Zhenhai Han

**Affiliations:** * College of Horticulture, China Agricultural University, Beijing 100193, P. R. China; † Zhongbaolvdu Agricultural Research Centre, Beidaihe, Hebei, 066100, China

**Keywords:** RNA-Seq, BSA-Seq, multi-omics, *Malus domestica* Borkh., *Botryosphaeria dothidea* (Moug. ex Fr.) Ces. & De Not

## Abstract

Apple fruit ring rot (FRR), caused by *Botryosphaeria dothidea*, is a worldwide disease that impacts Asian apple production regions. However, no substantial progress has thus far been made toward the mapping of candidate genes or the development of effective genetic makers. In this five-year study, the resistance of 1,733 F1 hybrids from the cross ‘Jonathan’ × ‘Golden Delicious’ was phenotyped by non-wounding inoculation with four *B. dothidea* isolates. We first conducted systematic comparison of different analytic strategies for bulk segregant analysis by re-sequencing (BSA-Seq) and obtained suitable one for outbreeding species such as *Malus*. Forty-six quantitative trait loci (QTL) for resistance/susceptibility to the four isolates, including one QTL ‘hotspot’ on chromosome 14, were identified via BSA-Seq. Using integrated multi-omics strategies including RNA-sequencing, parental re-sequencing, BSA-Seq and meta-analysis of RNA-sequencing, fifty-seven candidate genes and corresponding functional mutations from the QTL were predicted. Functional mutations located on the candidate genes were validated using kompetitive allele-specific PCR in hybrids and *Malus* germplasm accessions with extremely resistant/susceptible phenotypes. Ten effective markers for apple ring rot were developed. The results provide an example of rapid candidate gene mapping for complex traits in outbreeding species.

Apple fruit ring rot (FRR), caused by *Botryosphaeria dothidea*, is a worldwide disease that severely impacts Asian apple production regions (Guo *et al.* 2009). The phenotype of FRR incidence is qualitatively dominant against non-incidence, and the variation in susceptibility to FRR is attributed to the segregation of three major gene loci (Zhuang *et al.* 2011). Thirty-four major gene loci and six quantitative trait loci (QTL) for bot canker and FRR resistance/susceptibility have been genetically mapped on the apple genome (Cui *et al.* 2014). However, these QTL regions are too large for identifying candidate genes or to be effectively used in marker-assisted selection.

Bulk segregant analysis (BSA) is a powerful method for identifying DNA markers that are tightly linked to the causal genes in inbreeding plant species (Michelmore *et al.* 1991). Markers for both qualitative and quantitative trait loci can be identified by BSA, and BSA has been extensively used in cereal crops, vegetables, ornamentals, and some tree species (Lu *et al.* 2014; Pandey *et al.* 2017; Ries *et al.* 2016; Singh *et al.* 2016; Takagi *et al.* 2013; Wang *et al.* 2017; Win *et al.* 2017; Yang *et al.* 2013). BSA has been greatly improved in recent years by next-generation sequencing (NGS) technology, defined as BSA-Seq or QTL-Seq, and has been effectively used for QTL mapping in many plant species (Geng *et al.* 2016; Illa-Berenguer *et al.* 2015; Lu *et al.* 2014; Pandey *et al.* 2017; Ries *et al.* 2016; Singh *et al.* 2016; Takagi *et al.* 2013; Win *et al.* 2017).

Integrated multi-omics analysis can significantly enhance the efficient identification of candidate genes from QTL regions. Deep re-sequencing is an effective and reliable technology for detecting genome-wide genetic variations among cultivars (Kunihisa *et al.* 2016; Xing *et al.* 2016; Zhang *et al.* 2014). The transcriptome can reveal both the differentially expressed genes associated with biological processes and the genetic variations in coding sequences (CDS) (Balan *et al.* 2018; Bonnet *et al.* 2017; Hill *et al.* 2013). Large online transcriptome datasets are now available, and the meta-analysis of RNA-sequencing (RNA-Seq) datasets can provide an important reference for characterizing gene functions (Balan *et al.* 2017; Balan *et al.* 2018; Guo *et al.* 2017). For instance, copy number variation in a gene cluster encoding endopolygalacturonase, which mediates flesh texture and stone adhesion, was identified in peach using re-sequencing and RNA-Seq technology following traditional QTL mapping analysis (Gu *et al.* 2016). The biosynthesis, regulation, and domestication of bitterness in cucumber were determined using integrated approaches, including genome-wide association studies (GWAS), re-sequencing, and transcriptome analysis (Shang *et al.* 2014).

In this study, we integrated whole genome parental re-sequencing, BSA-Seq, and RNA-Seq meta-analysis to identify QTL and screen candidate genes for FRR resistance/susceptibility. Markers in the regions of the candidate genes were validated by kompetitive allele-specific PCR (KASP).

## Materials and Methods

### Plant material

*Malus* germplasms (60) and biparental cross hybrids (1,773) (‘Jonathan’ × ‘Golden Delicious’) were used as segregating populations. The hybrid cross was performed in 2002 and the seedlings were planted in 2003. All plant materials were subjected to conventional cultivation management practices.

### Phenotyping and sampling

In the years of 2008, 2009, 2014, 2015, and 2016, phenotyping for FRR resistance was undertaken under room temperature conditions using the ripened fruit and mycelia of four *B. dothidea* isolates (Zz026, Ls1, Lw023, and Lw048) (Guan *et al.* 2015; Zhuang *et al.* 2011).

### Re-sequencing of ‘Golden Delicious’ and ‘Jonathan’ and data processing

Genomic DNA was extracted from the leaves of ‘Golden Delicious’ and ‘Jonathan’ using a Genomic DNA Isolation Kit (TianGen, Beijing, China). The Illumina sequencing libraries were constructed using NEBNext DNA Library Prep Master Mix. Paired-end sequencing was performed using the Illumina HiSeq2500 sequencer (Illumina, San Diego, CA). Burrows-Wheeler Aligner (BWA) was used to map clean reads to the apple double haploid (DH) genome (Li and Durbin 2009; Daccord *et al.* 2017). SNP and InDel calling were conducted using SAMtools (Li *et al.* 2009; Li 2011). SNPs and InDels with a lowest phred-scaled quality of 30 and a minimum read depth of 10 were kept. Structure variation calling was conducted using the SpeedSeq pipeline (Chiang *et al.* 2015). InterproScan5.21was used to conduct functional domain analysis of all the genes in the apple genome (Zdobnov and Apweiler 2001). *Cis*-acting regulatory elements in upstream of the genes were analyzed using PlantCARE web tools (Rombauts *et al.* 1999). Finally, all the variations were annotated with ANNOVAR based on the functional domain, *cis*-acting regulatory elements, and gene model information (Wang *et al.* 2010).

### DNA pooling and BSA data processing

The resistant/susceptible bulks were constructed using 1,773 hybrids of ‘Jonathan’ × ‘Golden Delicious’ (Guan *et al.* 2015; Zhuang *et al.* 2011). Based on the lesion length data after fruit non-wounding inoculation with four isolates for five years, hybrids exhibiting the non-incidence phenotype in at least two years were included in the resistance (R) bulk, while incident hybrids with random lesion lengths in at least two years were chosen as the susceptible (S) bulk. Genomic DNA was extracted from the leaves of the selected hybrids using a Genomic DNA Isolation Kit (TianGen). Eight DNA libraries were constructed after DNA pooling based on the group information. The method of Genomic DNA extraction and the DNA library construction were the same as above. Paired-end sequencing was performed using the Illumina HiSeq X Ten platform (Illumina). The clean reads were mapped to the apple DH genome using BWA, and only uniquely mapped reads with a minimum phred-scaled map quality score of 20 were kept.

The SNPs showing polymorphisms between parents or which were double-heterozygous in the two parents were subdivided into three subsets: J-type, whereby the genotype of the markers is homozygous in ‘Golden Delicious’ and heterozygous in ‘Jonathan’; G-type, whereby the genotype of the markers is heterozygous in ‘Golden Delicious’ and homozygous in ‘Jonathan’; and H-type, whereby the genotype of the markers is heterozygous in both parents.

Read counts with different genotypes from extremely resistant and susceptible pools were extracted and separated based on the G-, J-, and H-type subsets, respectively. Thus, for each isolate, three files (G, J, and H) with read counts of the coupled pools were generated.

The allele frequency difference (AFD) between two coupled pools and G-value in each site were calculated based on the read counts. Nadaraya-Watson kernel regression was used as a smoothing function, and the G’-value at each site within the 1 Mbp sliding window size was calculated (Magwene *et al.*, 2011). The significance threshold of G’ was estimated using an empirical approach proposed by Magwene *et al.* (2011). Significant regions with false discovery rate (FDR) <0.01 were selected as candidate QTL.

The QTL analysis was performed three times using each of the three subsets of data. The QTL could be mapped to the pollen, maternal, or both parents using the marker data subset G, J, or H, respectively. QTL with peak G’ value exceeding 30% of the significant threshold were defined as major QTL.

### Estimating the impact of the reference genome on QTL detection

To investigate the impact of the reference genome on QTL detection, the clean sequencing reads from the two bulked DNA pools (resistant/susceptible to isolate Zz26) were processed to detect QTL using two versions of the apple reference genome (diploid version 1.0p and DH) (Velasco *et al.* 2010; Daccord *et al.* 2017). The DNA sequences without gaps (Ns) of the QTL were obtained from the corresponding genome (diploid version 1.0p or DH) using SAMtools software (Li *et al.* 2009; Li 2011). The LASTZ sequence alignment program was used to align the sequences against the genome, and dot plots were visualized in the R statistical environment (Harris 2007). To verify the reliability of the QTL, 3-5 markers were selected from each QTL region, and all individuals in the two bulks were genotyped using the KASP assay. Chi-square tests of the genotypic frequency of a certain marker indicating significant differences (*P*-value <0.05) between the two bulks were considered reliable.

### Comparison of different statistical methods for QTL identification

To determine the best statistical method, we compared the SNP index (SI), Euclidean distance (ED), and the G-value (GV) methods for QTL identification using the phenotype data of resistance/susceptibility to the Zz26 isolate. The absolute value of the △SNP index (|△SI|) was used for SI (Wang *et al.* 2017). Where large numbers of SNPs exist, fitting the ED using a Loess curve with a polynomial exponent of one in the MMAPPR may be hugely time-consuming and computationally intensive (Hill *et al.* 2013). Instead, we used Nadaraya-Watson kernel regression as a smoothing function in ED (Magwene *et al.* 2011). To get a fair decision of the three statistical methods, we adopted the same threshold selection method. The median of all the scores plus three-times the standard deviation (SD) were used as thresholds for SI, ED, and GV.

$$G^{\prime }=\sum_{j\;in\;W}k_{j}G_{j}$$ (1)

$$k_{j}=\frac{{\left(1-D_{j}^{3}\right)}^{3}}{\sum_{j}{\left(1-D_{j}^{3}\right)}^{3}}$$ (2)

$$ED=\sqrt{{\left(f_{A_{R}}-f_{A_{S}}\right)}^{2}+{\left(f_{T_{R}}-f_{T_{S}}\right)}^{2}+{\left(f_{G_{R}}-f_{G_{S}}\right)}^{2}+{\left(f_{C_{R}}-f_{C_{S}}\right)}^{2}}$$ (3)

$${\left(ED^{4}\right)}^{\prime }=\sum_{j\;in\;W}k_{j}{\left(ED^{4}\right)}_{j}$$ (4)

In the GV method, the G-value was calculated at each SNP using the standard G–statistic followed by smoothing using equation 1, where the sum includes all SNPs within the window W bracketing the SNP, and equation 2, where *D_j_* is the standardized distance, with value 0 at the focal position and value 1 at the edge of the window. Similarly, in the ED method, the Euclidean distance is first calculated at each SNP location using equation 3, where the letters represent the corresponding allele frequency of the bases in each pool. This distance is then raised to four-times the power, following which the data are fit using Nadaraya-Watson kernel regression, *i.e.*, equation 4. Also, for the SI method in this study, |△SI| was calculated at each maker and then smoothed using the same smoothing function.

### RNA-Seq library construction, sequencing, and data processing

The fruit of three hybrids randomly chosen from each of the extremely resistant/susceptible bulks were used for the RNA-Seq analysis (as three independent biological replicates). Three ripened apples from each hybrid were inoculated without wounding with the mycelia of the *B. dothidea* Zz26 isolate, and healthy flesh from the very edge of the lesion was cut and sampled at 0 h, 24 h, 48 h, and 96 h after inoculation (Guan *et al.* 2015). Three apples of each non-inoculated hybrid were also sampled as controls.

Total RNA was extracted using the modified CTAB method (Moser *et al.* 2004). For each sample, 5 μg total RNA was used to isolate mRNA for the preparation of an RNA-Seq library using NEBNext Poly(A) mRNA Magnetic Isolation Module and NEBNext Ultra Directional RNA Library Prep Kit for Illumina (New England Biolabs) following the manufacturer’s protocols. The cDNA library was sequenced (paired-end 150) from both the 5′ and 3′ ends on the Illumina HiSeq X Ten platform (Illumina) according to the manufacturer’s instructions. The RNA-Seq reads were mapped to the apple DH genome with HISAT2 (Kim *et al.* 2015). StringTie was used to conduct transcript assembly and quantification (Pertea *et al.* 2015).

DESeq2 software was used to detect differentially expressed genes (DEGs) between the resistant and susceptible hybrids (Love *et al.* 2014). Blast2GO was used for gene ontology (GO) classification and GO enrichment analysis (Conesa *et al.* 2005). KOBAS2.0 was used for Kyoto Encyclopedia of Genes and Genomes (KEGG) metabolism annotation and KEGG enrichment analysis (Xie *et al.* 2011). The .bam files from one individual hybrid were merged, followed by genotyping by SAMtools (Li *et al.* 2009; Li 2011). SNPs and InDels were called and clustered between resistant and susceptible hybrids.

### Candidate gene mining From the QTL regions based on multi-omics data

#### Narrowing down the QTL regions with varied sliding windows sizes:

By using the BSA-Seq data, all markers located on the gene body or up-stream regions of genes were selected from ± 0.5 Mb regions of the QTL main peak and sub-peaks, if present. The markers were then filtered, and those makers with a G-value above the G’-value were selected for subsequent analysis. Sliding window analysis with 0.125 Mbp, 0.25 Mbp, 0.5 Mbp, 0.75 Mbp, and 1 Mbp window sizes was used. The standard deviation and mean of those peaks were calculated to measure the stability of the peak position. Overlapped peak regions with different window sizes were considered to be the defined region

#### Excluding genes from QTL intervals using the parental re-sequencing data:

Too many genes were present in the narrowed-down QTL intervals. To further reduce the gene number, another criterion for candidate functional single nucleotide variants (SNVs) and structural variations (SVs) screening is that all the variations must be consistent with Mendelian inheritance; *i.e.*, if the QTL is located only in the pollen parent, then the genotype of the screened variant in the pollen parent must be heterozygous, the genotype in the maternal parent must be homozygous, and vice versa. By using the parental SNV and SV database, many genes within the narrowed-down QTL intervals were culled, except where upstream (2,000 bp) variations between the two parents may affect *cis*-element functioning, or where exonic variations between parents may cause stop-gain, frameshift, or domain non-synonymous altering.

#### Excluding genes from candidates using transcriptome data:

Gene transcription is often affected by variations in up-stream regions, especially on the *cis*-elements (Espley *et al.* 2009; Kobayashi *et al.* 2004; Zhang *et al.* 2017). The exonic variation can be also detected in the corresponding cDNA samples if the gene is transcribed (Han *et al.* 2018; Piskol *et al.* 2013). By using transcriptomic data, the unassociated genes can be further culled from the above-mentioned candidate genes, including those absent in the unigene dataset and thus un-transcribable with those variations in upstream *cis*-elements, but absent in the DEG dataset; and those with exonic variations absent in the unigene mRNA SNP/InDel-calling dataset.

#### Excluding genes from candidates with functional alterations in the incorrect bulk pool:

We attempted to assemble haplotype blocks using paired-reads. The Hidden Markov Model-based algorithm integrated in SAMtools was adopted to assemble haplotype blocks from the DNA sequence reads (Li *et al.* 2009; Li 2011). The haplotype blocks from the resistant/susceptible bulks were considered as main haplotype blocks representing the haplotype shared by most of the samples in the bulks. BSA markers were used as ‘anchor markers’ to assign the allele frequency to the haplotype. Thus, the haplotype blocks could be determined as enriched in resistant or susceptible pools based on the BSA makers.

### Marker validation and gene expression analysis

To examine the RNA-Seq data, randomly selected genes were validated using real-time quantitative (RT-q)PCR, and the gene-specific primers were designed with primer premier software (version 5.0) (Premier Biosoft Interpairs, Palo Alto, CA) (Table S1). One microgram of total RNA was used in the reverse transcription in a total volume of 20 μL in the presence of 6-mer random primer and oligo primer according to the protocol of the TaKaRa kit (TaKaRa Biotechnology Co., Ltd., Japan). The PCR reactions were run in three replicates in a Bio-Rad Sequence Detection System (Bio-Rad Life Science Research, Hercules, CA, USA). *Actin* was chosen as an internal control gene for normalization. Quantifying the relative expression of the genes at the three sampling time points was performed using the delta-delta Ct method (Livak and Schmittgen 2001). All data were expressed as the mean ± SD after normalization.

### Validation of SNPs and InDels

Primers were designed using Primer-blast software (NCBI, Maryland, USA) based on the 200-bp sequences flanking the SNPs and InDels to be validated. Then, monoclonal PCR products comprising SNP loci of interest were amplified in both parents and sequenced using Sanger sequencing (BGI, Beijing, China) to verify the SNPs obtained from the re-sequencing data between the parents.

### KASP genotyping assay and statistical analysis

A total of 110 DNA samples, including 20 and 30 individuals in the bulks resistant and susceptible to isolate Zz26, respectively, and also 60 *Malus* germplasm accessions were genotyped using the KASP assay (LGC Genomics, Beverly, MA, USA). The primers were designed based on the 200-bp sequence flanking the SNPs (Table S2). The DNA of ‘Jonathan’ and ‘Golden Delicious’ was used as control. Then the genotypes were validated by Sanger sequencing. Data were analyzed using the “Endpoint Genotyping” method of the Light Cycler 480 Software (release 1.5.0).

The segregation biases of markers between resistant and susceptible bulks were tested by Chi-square test. Chi-square test was run twice, the first, allele A was supposed to be completely/partially dominant on B, and the next, allele B completely/partially dominant on A. The scenario which Chi-square value was statistically significant was accepted.

### Data Availability

The RNA-sequencing data and the whole genome re-sequencing data of the pooled DNA from the bulks and un-pooled DNA from the parental cultivars have been deposited in the NCBI Sequence Read Archive (SRA) with the accession number PRJNA392908. Gene expression data and other additional datasets and codes are also available at Figshare and File ‘Readme.txt’ contains detailed description of all the additional datasets. File S1 contains all the detailed descriptions of supplemental materials. Supplemental material available at Figshare: https://doi.org/10.25387/g3.7819688.

## Results

### Bulk construction and whole genome re-sequencing

**Figure 1:**
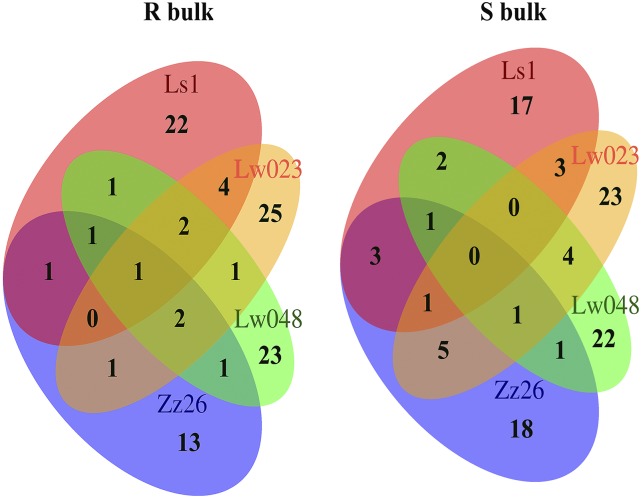
Venn diagrams of the hybrid numbers (‘Jonathan’ × ‘Golden Delicious’) included in the bulks with phenotypes of extremely resistant (R) and susceptible (S) to apple fruit ring rot (*Botryosphaeria dothidea*) isolates Ls1 (red), Lw023 (orange), Lw048 (green) and Zz26 (blue).

Based on the phenotypes over five years (2008, 2009, 2014, 2015, and 2016), eight bulks were developed, each including 20∼37 hybrids with extremely resistant or susceptible phenotypes to each of the four *B. dothidea* isolates (Zz26, Ls1, Lw023, and Lw048). A total of 199 hybrids were included in the eight bulks, of which 17 and 21 exhibited extreme resistance or susceptibility to at least two pathogen isolates, respectively (Figure 1 and Table S3).

The whole genome re-sequencing data of the pooled DNA from the bulks and un-pooled DNA from the parental cultivars, ‘Jonathan’ and ‘Golden Delicious’, have been deposited in the NCBI Sequence Read Archive (SRA) with the accession number PRJNA392908. A total of 163,934,426 and 172,856,412 clean reads were obtained by parental re-sequencing for ‘Golden Delicious’ and ‘Jonathan’, respectively (Table S3). About 329.4 M reads were mapped to the apple DH genome (Daccord *et al.* 2017), of which about 207.5 M reads were uniquely mapped (Table S3). SNVs and SVs called and annotated using the parental re-sequencing data are included in Table S4–S7. The overall consistency between the NGS and Sanger sequencing platforms was 99.9% and 85% for SNVs and SVs, respectively (Table S8 and Table S9). Based on the re-sequencing data of the two parents, a total of 1,126,610 J-type SNPs, 500,397 G-type SNPs, and 3,098,152 H-type SNPs were identified (Table 1).

**Table 1:** Numbers of different types of markers used in bulk segregant analysis

Isolate	J-type	G-type	H-type	Sum
J *vs.* G	1126610	500397	3098152	4725159
Zz26	1475502	545025	1272274	3292801
Ls1	703321	257515	732935	1693771
Lw023	1013974	381292	961013	2356279
Lw048	1538283	535547	1433517	3507347

Notes: G-type: the genotype of the marker is heterozygous in ‘Golden Delicious’ and homozygous in ‘Jonathan’; J-type: the genotype of the marker is homozygous in ‘Golden Delicious’ and heterozygous in ‘Jonathan’; H-type: the genotype of the marker is heterozygous in both parents;J *vs.* G:all the numbers of the three types of markers based on the genotype of ‘Golden Delicious’ and ‘Jonathan’.

Sequencing of the eight bulks generated a total of 917,367,968 reads. The sequence reads of the bulked samples provided on average 39.6–60 X coverage for the DH apple genome (Table S3). For all the reads (937,097,528) from the eight bulks, 97.89% (917,367,968) were mapped to the reference genome and 72.8% (682,727,645) were uniquely mapped (Table S3).

After filtering, the sum of the remaining J-, G-, and H-type SNPs ranged from 1,693,771 to 3,507,347, and the corresponding SNP density across the genome ranged from 2601/Mb to 5387/Mb in the extreme bulks of the four isolates (Table 1). The distribution of SNPs in the genome, shown as the number of SNPs per Mb with a 10 Kbp sliding window, was not even, and almost all regions in the genome excluding the gaps were covered by the sliding window analysis (Figure S1–S4).

### Comparison of statistical methods for QTL identification

The profiles of the corresponding major QTL (J06.1, J08.1, J10.1, J10.2, J10.3, G11.1, G15.1, H03.1, H14.1, H14.2, and H16.1) identified through SI, ED, and GV coincided or overlapped well, indicating that the effects of the major QTL were relatively robust. For all statistical methods, the effects of noise were effectively reduced and AFD was measured between the two extreme pools (Figure S5, Figure S6 and Figure S7). However, the QTL by SI covered broader regions and were less significant than by ED or GV (Figure S5, Figure S6, Figure S7 and Table 2), implying that the QTL sensibility of SI was comparatively lower. As a consequence, one reliable minor QTL (G02.1-GV) was missed in SI (Figure S7 and Table 2). Distinct unbiasedness and low false positives in the QTL were achieved using GV, especially for H-type QTL. The QTL intervals by GV (2,551,554 bp) were much narrower than that by ED (2,618,442 bp) (Table 2). Additionally, some false positive QTL were found in ED (Figure S6 and Table S11). Collectively, GV was the best statistical method and was thus explored subsequently.

**Table 2:** Comparison of overlapped QTL for apple (Jonathan × Golden Delicious) resistance/susceptibility to Botryosphaeria dothidea isolate Zz26, detected by three statistic methods

Statistic method	QTL	Chromosome	Origin	QTL start	QTL end	QTL region	Peak position	Peak (SI/G’/ED)	Threshold	Percentage exceeding threshold	KASP confirmed (YES/NO)
GV	H03.1	Chr03	H	33952320	35776974	1824654	34822605	5.2269	3.8659	35.21%	YES
SI	H03.1	Chr03	H	33397901	35869112	2471211	35203353	0.1973	0.1700	16.07%	YES
ED	H03.1	Chr03	H	34041631	35889232	1847601	35389232	0.017	0.0121	40.43%	YES
ED	J06.1	Chr06	J	1501802	7049918	5548116	4800068	0.0238	0.0076	211.49%	YES
GV	J06.1	Chr06	J	1501802	7049918	5548116	4800068	8.935	3.8199	133.91%	YES
SI	J06.1	Chr06	J	1668863	7529246	5860383	4799846	0.2276	0.1500	51.71%	YES
ED	J08.1	Chr08	J	10795726	11843155	1047429	11295726	0.0094	0.0076	23.60%	YES
GV	J08.1	Chr08	J	10795726	11795726	1000000	11295726	4.4068	3.8199	15.36%	YES
SI	J08.1	Chr08	J	10824897	11743561	918664	11295726	0.1644	0.1500	9.59%	YES
GV	J10.1	Chr10	J	25782140	27140011	1357871	26111263	6.6854	3.8199	75.02%	YES
ED	J10.1	Chr10	J	25781888	27279984	1498096	26668670	0.0127	0.0076	66.85%	YES
SI	J10.1	Chr10	J	25477302	27140011	1662709	26600792	0.1787	0.1500	19.13%	YES
ED	J10.2	Chr10	J	29538171	38527887	8989716	37748893	0.0187	0.0076	145.20%	YES
SI	J10.4	Chr10	J	30998909	38522983	7524074	37749874	0.2034	0.1500	115.00%	YES
GV	J10.4	Chr10	J	36744410	38505907	1761497	37748893	7.313	3.8199	91.45%	YES
SI	G11.1	Chr11	G	9782493	11021296	1238803	10408719	0.1641	0.1400	17.24%	YES
ED	G11.1	Chr11	G	9782493	11038906	1256413	10408719	0.008	0.0055	45.00%	YES
GV	G11.1	Chr11	G	9908719	10949757	1041038	10408719	4.5433	3.5046	29.64%	YES
ED	H14.1	Chr14	H	14647346	17683075	3035729	15269958	0.0219	0.0121	80.77%	YES
GV	H14.1	Chr14	H	14685326	17283075	2597749	15466864	5.919	3.8659	53.11%	YES
SI	H14.1	Chr14	H	14856674	17289065	2432391	15466864	0.2052	0.1700	20.68%	YES
SI	H14.3	Chr14	H	25112559	26112559	1000000	25612559	0.2373	0.1700	39.61%	YES
ED	H14.2	Chr14	H	25112559	26436147	1323588	25612559	0.0355	0.0121	193.23%	YES
GV	H14.2	Chr14	H	25112559	26295711	1183152	25612559	7.4029	3.8659	91.49%	YES
ED	G15.2	Chr15	G	51474032	53363776	1889744	51974032	0.0109	0.0055	99.46%	YES
GV	G15.1	Chr15	G	51484641	53158477	1673836	51984641	4.9519	3.5046	41.30%	YES
SI	G15.1	Chr15	G	51659034	54930321	3271287	52514030	0.1695	0.1400	21.11%	YES

Notes: Statistic method, GV: G value method, SI: SNP index method, ED: Euclidean distance method.

### The impact of the reference genome on QTL detection

**Figure 2:**
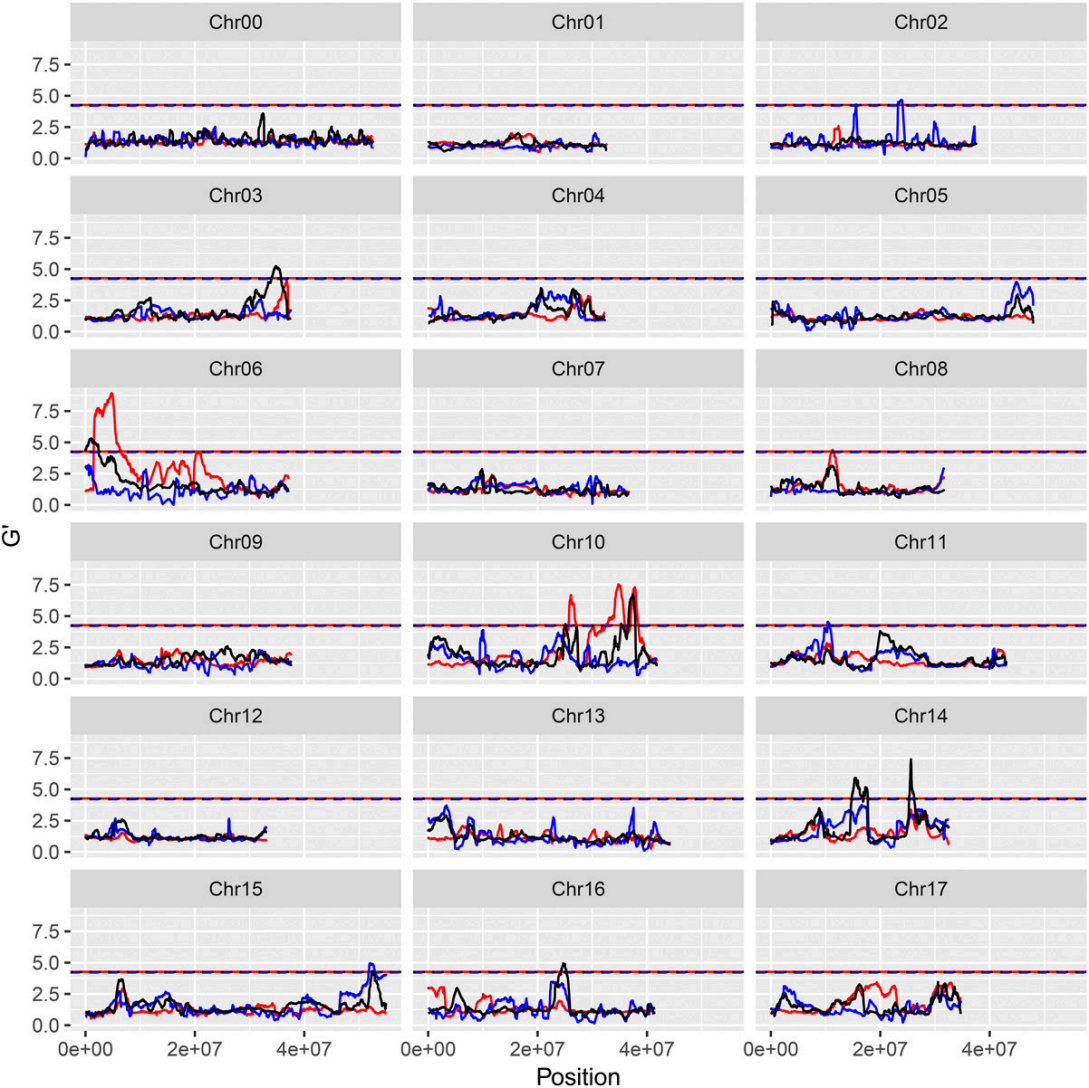
G’ value profile of the QTL for apple (‘Jonathan’ × ‘Golden Delicious’) resistance/susceptibility to *Botryosphaeria dothidea* isolate Zz26. Y-axis represents G’ value. X-axis represents chromosome position. Red lines: ‘Jonathan’, blue lines: ‘Golden Delicious’, black lines: ‘Golden Delicious’ & ‘Jonathan.’

By using the diploid (version 1.0p) and DH genomes, 14 and 12 significant QTL were identified, respectively (Figure 2 and Table S10). The seven reliable QTL in the DH genome coincided or overlapped precisely with nine reliable QTL from the diploid genome (Table S13). However, five reliable QTL (G02.1, J08.1, H16.1, H14.1, and H14.2) in the DH genome did not match any QTL from the diploid genome (Table S13). Conversely, two reliable QTL (G09.1 and G09.2) identified by using the diploid genome were missing from the results of the DH genome (Table S13). Based on the sequence alignment, gaps were clearly detected in two QTL regions between the two genome versions (Table S13 and Figure S9). One QTL (J05.1) in the diploid genome was aligned to chromosome 10 of the DH genome and overlapped with one QTL (J.10.2) (Table S13, Figure S9, and Figure S10). These data confirmed the reliability of the QTL detection and also suggested that using DH genome should generally produce better results, but a few QTL may be potentially missed due to the loss of segments/regions during DH process and recombination.

### Identification of QTL for FRR resistance

**Figure 3:**
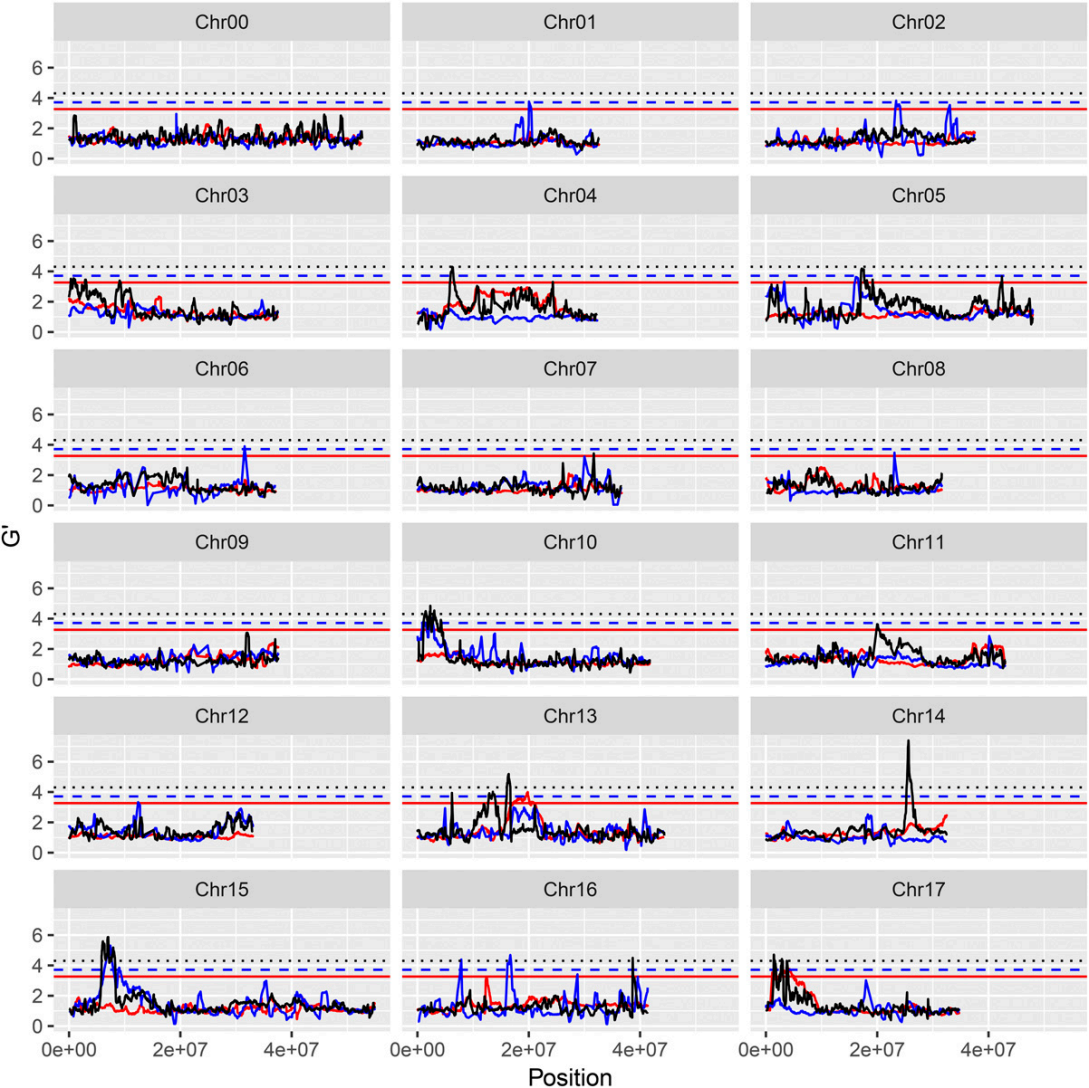
G’ value profile of the QTL for apple (‘Jonathan’ × ‘Golden Delicious’) resistant/susceptible to *Botryosphaeria dothidea* isolate Ls1. Y-axis represents G’ value. X-axis represents chromosome position. Red lines: ‘Jonathan’, blue lines: ‘Golden Delicious’, black lines: ‘Golden Delicious’ & ‘Jonathan.’

**Figure 4:**
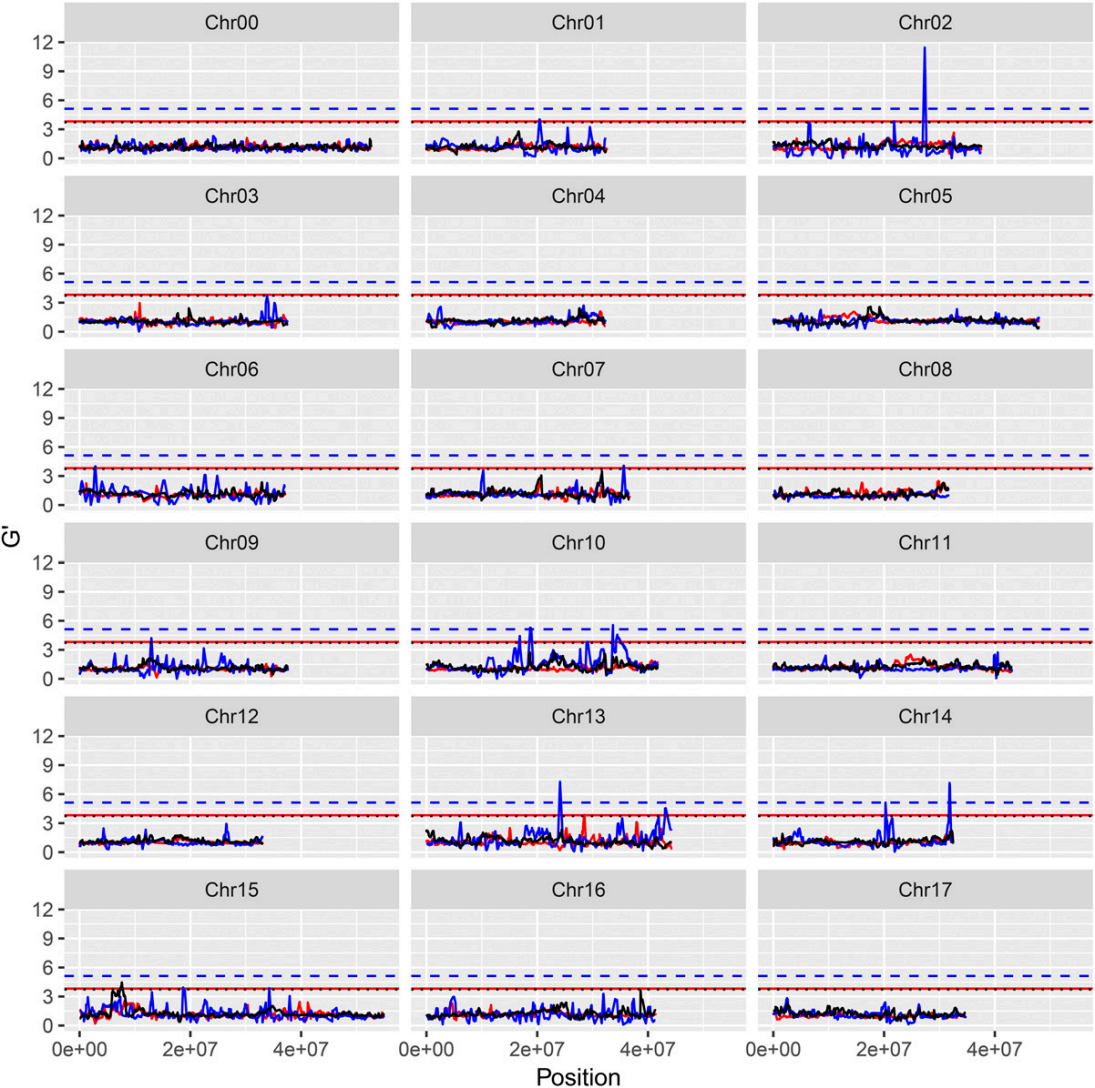
G’ value profile of the QTL for apple (‘Jonathan’ × ‘Golden Delicious’) resistant/susceptible to *Botryosphaeria dothidea* isolate Lw023. Y-axis represents G’ value. X-axis represents chromosome position. Red lines: ‘Jonathan’, blue lines: ‘Golden Delicious’, black lines: ‘Golden Delicious’ & ‘Jonathan.’

**Figure 5:**
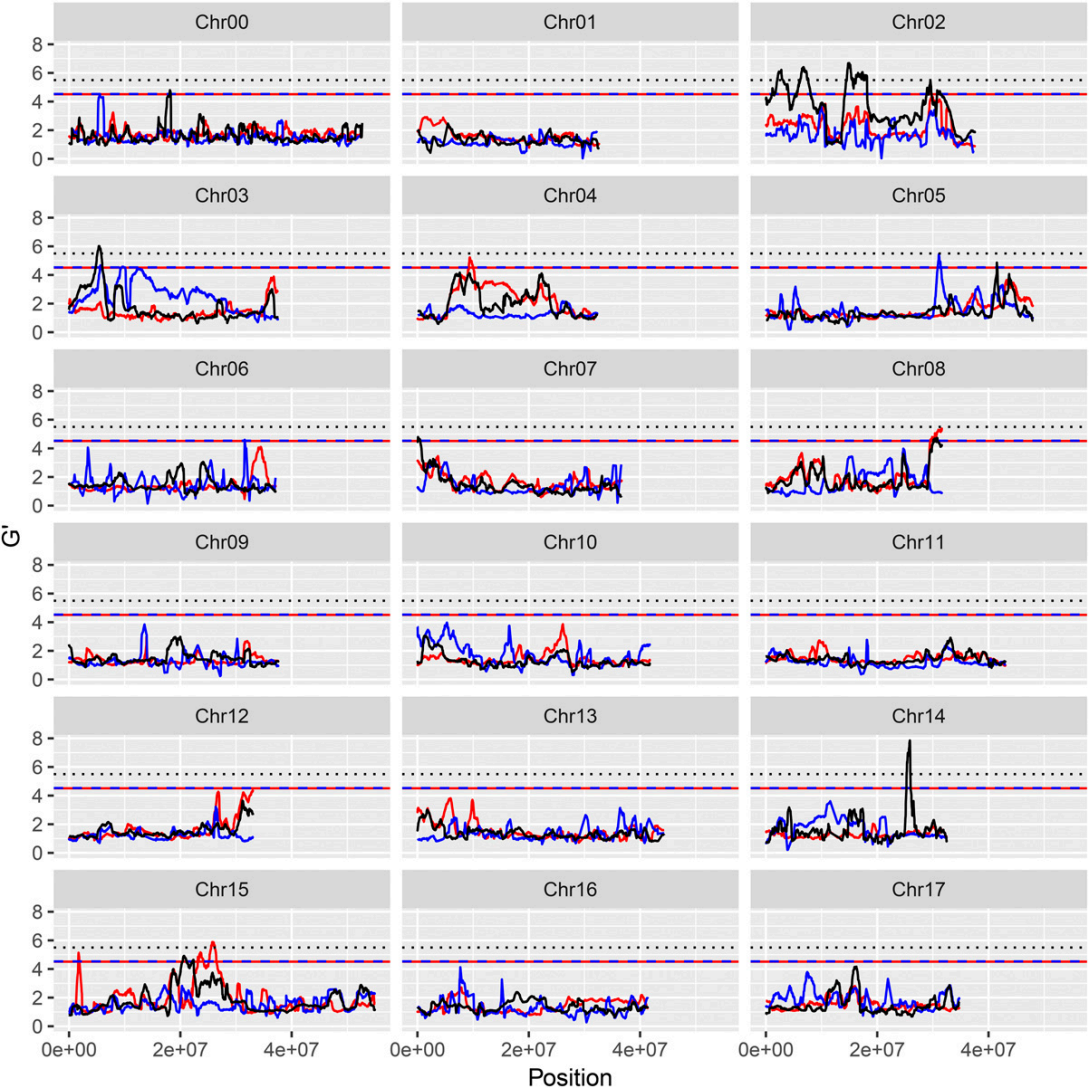
G’ value profile of the QTL for apple (‘Jonathan’ × ‘Golden Delicious’) resistant/susceptible to *Botryosphaeria dothidea* isolate Lw048. Y-axis represents G’ value. X-axis represents chromosome position. Red lines: ‘Jonathan’, blue lines: ‘Golden Delicious’, black lines: ‘Golden Delicious’ & ‘Jonathan.’

For resistance/susceptibility to isolate Zz26, 12 significant QTL were obtained, six of which (J06.1-Zz26, J10.1-Zz26, J10.2-Zz26, J10.3-Zz26, H14.1-Zz26, and H14.2-Zz26) were considered as major QTL (Figure 2 and Table S10). For resistance/susceptibility to *B. dothidea* isolate Ls1, Lw023, and Lw048, 14, 8, and 12 significant QTL were detected, respectively. Of these QTL, seven (H14.1-Ls1, G15.1-Ls1; G02.1-Lw023, G13.1-Lw023, G14.2-Lw023, H14.1-Lw048, and J15.2-Lw048) constituted major QTL (Table S14 and Figures 2, 3, 4, and 5).

**Figure 6:**
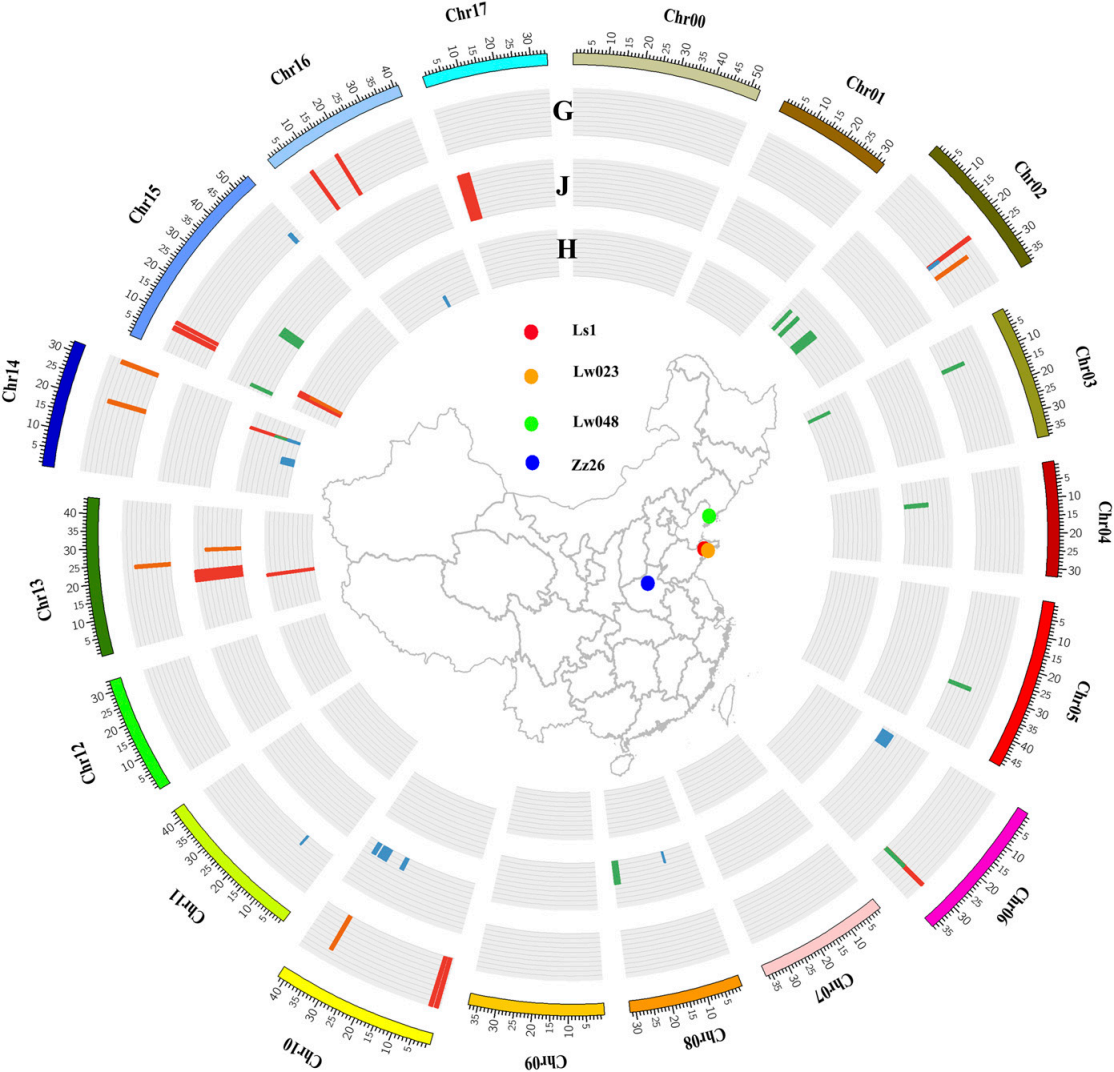
Circular overview of the QTL for apple resistance/susceptibility to fruit ring rot (*Botryosphaeria dothidea*) isolates Ls1 (red), Lw023 (orange), Lw048 (green), and Zz26 (blue) across the genome. The outer circle: QTL from ‘Golden Delicious’ (G); intermediate circle: QTL from ‘Jonathan’ (J); the inner circle: QTL from both ‘Golden Delicious’ and ‘Jonathan’. The colored dots in the central map indicate the geographical origin of the pathogen isolates

Of the significant QTL, five QTL regions coincided or overlapped with resistance/susceptibility to more than two isolates. The QTL G02.1-Zz26/G02.1-Ls1, G15.1-Ls1 / H15.1-Lw023 and G06.1-Ls1/G06.1-Lw048 coincided, while the QTL regions of J10.2-Zz26/G10.2-Lw023 and H14.2-Zz26/H14.1-Ls1/H14.1-Lw048 overlapped (Table S14 and Figure 6). H14.2-Zz26/H14.1-Ls1/H14.1-Lw048 were consistently mapped to the same region along with four previously reported major gene loci (sf-g14-ls1, sf-g14-lw048, Rb-g14-lw048, and Rb-g14-zz26) (Figure S11) (Cui *et al.* 2014).

### Narrowing down the QTL intervals with varied sliding window sizes

By using varied sliding window sizes, the 46 QTL for FRR resistance to the four pathogen isolates were significantly reduced (Table S15). The spanning regions of the 13 major QTL were narrowed down from 1,685,139 bp to 743,971 bp on average, and thus the significance of the average GV scores was also increased from 5.80 to 6.25 (Table S15). Several broader QTL intervals were split into two or more narrower sub-peaks; *e.g.*, the QTL J06.1-Zz26 was spliced into two sub peaks, J06.1_sub1-Zz26 and J06.2_sub2-Zz26, respectively (Table S15). As a result of this splicing, the QTL interval was narrowed down from 4,800,068 bp to 1,000,000 bp and 500,000 bp, and the average GV scores increased from 8.93 to 8.34 and 9.71, respectively (Table S15).

### Candidate gene mining from QTL regions based on multi-omics data

#### Excluding genes from the QTL intervals using parental re-sequencing data:

Of the 3,058 genes scattered on the 49 narrowed-down QTL regions including sub peaks (Table S15), 1,346 genes were by excluded using the parental SNV and SV databases, and 24,977 SNVs and 253 SVs were distributed in the 1,712 remaining candidate genes (Table S16).

#### Excluding genes from candidates using transcriptome data:

The RNA-Seq data were deposited in the NCBI Sequence Read Archive (SRA) with the accession number PRJNA392908. After removing low-quality reads and those derived from rRNA, the total reads input for mapping was 285.86 M, of which 207.9 M (80.63%) were aligned to the apple DH genome (Table S17). Overall, 136,983 transcripts from 86,808 genes (63,538 referenced and 23,270 non-referenced) were assembled. The Fragments Per Kilobase of transcript per Million mapped reads (FPKM) values of three representative genes, MD05G1236300, MD05G1261100, and MD08G1128800, were closely correlated with their relative expressions by RT-qPCR (R^2^ = 0.921-0.962, *P* < 0.01) (Figure S12). The correlation analysis and hierarchical cluster analysis revealed a close grouping of the same stage in both the extremely susceptible and resistant hybrids (Figure S13).

In total, 1,570 DEGs were detected between the extremely susceptible and resistant hybrids across the sampling time points (Figure S14). SNVs, including 503,067 SNPs and 37,402 InDels, were identified in 36,796 genes. Following functional annotation, 103,510 non-synonymous SNVs were identified and were distributed across 24,533 genes.

In addition to the transcriptomic data in this study, the RNA-Seq data of 25 transcriptome bio-projects and 432 samples from the SRA database were downloaded (Table S18). All the reads were mapped to the DH reference genome to quantify the expression of each gene. The expression data of the genes were also used to assist in the screening of organ-specific or non-expressive genes (Eide *et al.* 1996).

Using the transcriptomic data, 56 non-expressed genes and 46 non-DEGs without variations in their CDS were culled from the 503 candidates in the QTL intervals for resistance/susceptibility to isolate Zz26 (Table S16). In the QTL regions for resistance/susceptibility to isolate Ls1, Lw023, and Lw048, 221 of the 1,209 genes were excluded owing to non-expression in any apple fruit samples (Table S16).

#### Excluding genes from candidates by functional annotation:

One thousand and one of the 1,389 candidate genes were successfully assigned with Gene Ontology (GO) terms. Based on the detailed gene annotations, 386 genes were excluded due to the inconsistency of their organ/tissue/sub-cellular localization, developmental dynamics, and physiological pathway annotations, after which 1,003 genes remained (Table S16).

#### Excluding genes from candidates based on their functional alteration appearing in the incorrect bulk:

The allele frequency of the screened functional variations in the extremity pools can aid candidate gene prediction. However, only small parts of the SNPs used in the BSA analysis can be directly assigned with allele frequency. Here, we phased the variations and constructed 740,329 haplotype blocks in the two parents. BSA markers were used as ‘anchor markers’ to assign the AFD between two bulks to the haplotype. For the pollen parent ‘Golden Delicious’, 5,505,264 SNPs were phased into 364,768 haplotype blocks, and after anchoring, 162,184 (44.46%) haplotype blocks were assigned an AFD between bulks. For the maternal parent ‘Jonathan’, 6,122,520 SNPs were phased into 375,561 haplotype blocks, and 196,220 (52.25%) haplotype blocks were assigned an AFD to the bulk pools. Considering meiotic crossover, the haplotypes of the male and female parent were referenced to each other. As for resistance/susceptibility to isolate Zz26, in this study, 2,051 of the 3,211 variations in 278 candidate genes were assigned an AFD, and the 42 genes with unexpected AFD values between the two bulk pools were excluded. For example, a stop-gain variation was screened in the CDS of MD15G1419300 in QTL G15.1, which is annotated as ‘mitogen-activated protein kinase kinase kinase’ gene; however, the stop-gain variation was enriched in the R pool, which is inconsistent with its function. On the contrary, a stop-gain variation in the CDS of MD10G1288400 in J10.3, a serine/threonine-protein kinase-like protein, was enriched in the S pool, which was expected.

Ultimately, 57 candidate genes from 46 significant QTL associated with resistance/susceptibility to four *B. dothidea* isolates were selected for further experimental validation (Table 3 and Table S14).

**Table 3:** Summary of the candidate genes mined from the QTL for apple (‘Jonathan’ × ‘Golden Delicious’) resistance/susceptibility to *Botryosphaeria dothidea* isolates

Isolate	Chromosome	QTL	gene ID	R/S	Mutation type	Gene annotation
Lw048	Chr02	H02.1	MD02G1036600	S	frame-shift insertion	Protein kinase superfamily protein
Lw048	Chr02	H02.1	MD02G1040200	R	CREs & nonsynonymous SNV	Transcriptional corepressor LEUNIG
Lw048	Chr02	H02.2	MD02G1085900	—	frame-shift deletion	Ankyrin repeat family protein
Lw048	Chr02	H02.3	MD02G1170800	R	CREs	AP2/B3-like transcriptional factor family protein
Lw048	Chr02	H02.3	MD02G1174000	S	CREs & nonsynonymous SNV	Probable receptor-like serine/threonine-protein kinase
Lw048	Chr02	H02.3	MD02G1191600	S	stop-gain	Pathogenesis-related protein 1C
Lw048	Chr03	H03.1	MD03G1067300	S	nonsynonymous SNV	NB-ARC domain-containing disease resistance protein
Lw048	Chr06	G06.1	MD06G1173700	S	stop-gain	Serine/threonine-protein kinase ATM
Lw048	Chr08	J08.1	MD08G1229700	—	frame-shift insertion	Protein kinase family protein
Lw048	Chr08	J08.1	MD08G1239000	—	frame-shift insertion	Disease resistance family protein
Lw048	Chr08	J08.1	MD08G1241800	—	nonsynonymous SNV	Leucine-rich receptor-like protein kinase family protein
Lw048	Chr08	J08.1	MD08G1244800	S	stop-loss	Protein kinase superfamily protein
Lw048	Chr04	J04.1	MD04G1066000	S	stop-gain	CTR1_ARATH Serine/threonine-protein kinase CTR1
Lw048	Chr14	H14.1	MD14G1162500	—	CREs	Unknown
Lw048	Chr15	J15.3	MD15G1282900	S	stop-gain	Leucine-rich receptor-like protein kinase family protein
Ls1	Chr06	G06.1	MD06G1173700	S	stop-gain	Serine/threonine-protein kinase ATM
Ls1	Chr10	G10.1	MD10G1006400	S	stop-gain	WRKY transcription factor 19
Ls1	Chr10	G10.1	MD10G1007300	—	nonsynonymous SNV	Disease resistance protein
Ls1	Chr10	G10.1	MD10G1007600	S	nonsynonymous SNV	disease resistance protein
Ls1	Chr10	G10.1	MD10G1006500	—	nonsynonymous SNV	Disease resistance protein
Ls1	Chr10	G10.1	MD10G1006600	S	nonsynonymous SNV	Disease resistance protein
Ls1	Chr10	G10.1	MD10G1006800	—	nonsynonymous SNV	Disease resistance protein
Ls1	Chr10	G10.1	MD10G1006900	—	nonsynonymous SNV	Disease resistance protein
Ls1	Chr10	G10.2	MD10G1021000	S	frame-shift insertion	Disease resistance protein
Ls1	Chr13	H13.1	MD13G1191200	—	nonsynonymous SNV	PLAT/LH2 domain-containing lipoxygenase family protein
Ls1	Chr13	J13.1	MD13G1210700	—	CREs & nonsynonymous SNV	HR-like lesion-inducing protein-related
Ls1	Chr14	H14.1	MD14G1162500	—	CREs	Unknown
Ls1	Chr15	G15.1	MD15G1103400	—	nonsynonymous SNV	Transcriptional corepressor LEUNIG
Ls1	Chr15	G15.1	MD15G1104000	S	nonsynonymous SNV	Disease resistance protein
Ls1	Chr15	G15.1	MD15G1103200	—	nonsynonymous SNV	AIG2-like (avirulence induced gene) family protein
Ls1	Chr15	G15.2	MD15G1129100	—	frame-shift deletion	TOPLESS-related 1
Ls1	Chr17	J17.2	MD17G1028400	—	frame-shift deletion	Protein TPR2
Ls1	Chr17	J17.2	MD17G1028700	S	stop-gain	Ankyrin repeat-containing protein NPR4
Ls1	Chr17	J17.2	MD17G1029100	S	stop-gain	Ankyrin repeat family protein
Ls1	Chr17	J17.2	MD17G1029200	—	frame-shift deletion	Ankyrin repeat family protein
Ls1	Chr02	G02.1	MD02G1213800	S	nonsynonymous SNV	Pentatricopeptide repeat-containing protein
Ls1	Chr17	J17.2	MD17G1054100	—	nonsynonymous SNV	WRKY DNA-binding protein 3
Lw023	Chr02	G02.1	MD02G1229600	S	upstream	Two-component response regulator ORR22
Lw023	Chr02	G02.1	MD02G1230200	—	upstream	Seven transmembrane MLO family protein
Lw023	Chr10	G10.1	MD10G1111400	S	CREs & nonsynonymous SNV	Basic form of pathogenesis-related protein 1
Lw023	Chr10	G10.2	MD10G1243000	S	nonsynonymous SNV	WRKY transcription factor 14
Lw023	Chr10	G10.2	MD10G1243400	R	upstream	Receptor-like serine/threonine-protein kinase ALE2
Lw023	Chr14	G14.2	MD14G1236800	—	nonsynonymous SNV	HCP-like superfamily protein
Lw023	Chr14	G14.2	MD14G1236600	—	nonsynonymous SNV	Prostaglandin E synthase 2
Lw023	Chr15	H15.1	MD15G1106800	S	stop-gain	Leucine-rich repeat protein kinase family protein
Lw023	Chr15	H15.1	MD15G1103700	S	stop-loss	Disease resistance protein
Lw023	Chr15	H15.1	MD15G1104000	S	frame-shift deletion	Disease resistance protein
Lw023	Chr15	H15.1	MD15G1106600	—	CREs & nonsynonymous SNV	WRKY DNA-binding protein 11
Zz26	Chr02	G02.1	MD02G1213800	S	nonsynonymous SNV	Pentatricopeptide repeat-containing protein
Zz26	Chr11	G11.1	MD11G1116700	S	CREs	Ankyrin repeat-containing protein
Zz26	Chr15	G15.1	MD15G1416500	S	stop-gain	Disease resistance protein
Zz26	Chr03	H03.1	MD03G1259600	—	CREs	Serine/threonine-protein kinase
Zz26	Chr14	H14.2	MD14G1162500	—	CREs	Unknown
Zz26	Chr16	H16.1	MD16G1236700	R	CREs	Serine/threonine-protein kinase
Zz26	Chr06	J06.1_sub1	MD06G1017400	S	CREs	F-box/kelch-repeat protein
Zz26	Chr06	J06.1_sub2	MD06G1037600	S	CREs	NDR1/HIN1-like 8
Zz26	Chr08	J08.1	MD08G1120500	S	stop-gain	Pentatricopeptide repeat-containing protein
Zz26	Chr08	J08.1	MD08G1120700	S	frame-shift	Pentatricopeptide repeat-containing protein
Zz26	Chr10	J10.1	MD10G1169200	—	CREs	Kinase-like protein TMKL1
Zz26	Chr10	J10.2	MD10G1255900	S	stop-gain	F-box/kelch-repeat protein
Zz26	Chr10	J10.3	MD10G1288400	—	frame-shift deletion	Serine/threonine-protein kinase-like protein
Zz26	Chr10	J10.3	MD10G1288500	—	nonsynonymous SNV	Receptor-like protein kinase HSL1

Notes: Enriched pool (R/S/—): the enriched pools of the candidate functional variations, R: extremely resistant pools, S: extremely susceptible pools; —: unknown; CREs: *cis*-acting regulatory elements.

### Experimental validation of the candidate genes

The presence/absence of variations in the 14 candidate genes from 11 major QTL linked to resistance to FRR isolate Zz26 was confirmed by Sanger sequencing (Table S19 and Table S20). The segregation of the variants was then validated by KASP genotyping in hybrids with extreme phenotypes, and all the variants exhibited significant associations between genotypes and phenotypes (*P* < 0.05) (Table S21). The presence/absence of the markers was then verified in each 30 *Malus* germplasm accessions highly resistant/susceptible to FRR isolate Zz26 (Table S22). Ten KASP markers from nine QTL exhibited significant associations with the phenotypes (Table S23).

Of the 14 candidate genes, five genes (MD03G1259600, MD16G1236700, MD10G1169200, MD10G1288400, and MD10G1288500), located in four QTL (H03.1, H16.1, J10.1, and J10.3), constituted protein kinases or kinase-like proteins (Table S19). The variations were validated by Sanger sequencing in the *cis*-element or transcription factor binding site of the promotors of MD03G1259600, MD16G1236700, and MD10G1169200 (Table S20), which is highly consistent with the finding that the expressions of these genes in fruit from the resistant bulk 48 h after inoculation were significantly higher than that in the susceptible bulk (Figure S15). Frame-shift deletions and stop-gain mutants were located in the CDS of the other two protein kinase genes, MD10G1288400 and MD10G1288500, respectively, which may cause functional loss of the genes (Table S20).

From the major QTL J06.1, MD06G1037600, annotated as ‘NDR1/HIN1-like 8’ was ultimately screened as a candidate gene (Table S19). Re-sequencing and Sanger sequencing confirmed the variations in the *cis*-element and transcription factor (TF) binding site of this gene between parents (Table S20). The KASP analysis of the maker (KASP100) located in the promoter of MD06G1037600 exhibited significant association with the phenotype in the bulks (χ2= 7.4108 *P* = 0.006480 <0.05) and the 60 accessions with extreme phenotypes (χ2 = 5.93, *P* = 0.0146 <0.05) (Table S21, Table S23, and Table S20). The expression of this gene in the fruit from the resistant bulk 48 h after inoculation was consistently significantly higher than that in the susceptible bulk (Figure S15).

Three pentatricopeptide repeat-containing genes, MD02G1213800, MD08G1120500, and MD08G1120700, were identified as candidate genes from QTL G02.1 and J08.1 (Table S19). Nonsynonymous SNVs were distributed in the functional domain of MD02G1213800, and the KASP analysis of one functional variation marker (KASP146) exhibited significant association with the phenotype of the two extreme pools (χ2 = 9.736, *P* = 0.001807 <0.05) (Table S18 and Table S21). Stop-gain and frame-shift variations were detected and validated in both MD08G1120500 and MD08G1120700 (Table S19 and Table S20). Furthermore, KASP analysis confirmed that the stop-gain mutation A (KASP107), enriched in the susceptible bulk, exhibited significant AFD values between the two pools (χ2= 5.333, *P* = 0.02090 <0.05) and the 60 extreme accessions (χ2= 4.593, *P* = 0.0321 <0.05) (Table S20, Table S21, and Table S23). At the same time, frame-shift variations (KASP117) were also significantly enriched in the susceptible bulk (χ2 = 3.8595, *P* = 0.04950 < 0.05) and extremely susceptible accessions (χ2 = 4.0074, *P* = 0.0453 <0.05) (Table S20, Table S21, and Table S23).

In the region of QTL G15.1, a disease-resistance gene (MD15G1416500) was detected with the largest G’ value (Table S19). A functional SNP (C to A) causing stop-gain was present in its CDS, and allele A was enriched in the susceptible bulk (Table S19). Furthermore, the corresponding KASP maker (KASP66) was significantly associated with the phenotype in both the resistant/susceptible pools (χ2 =9.145, *P* = 0.0025 <0.05) and 60 extreme accessions (χ2= 5.079, *P* = 0.0242 <0.05) (Table S20, Table S21, and Table S23).

Two F-box/kelch-repeat proteins (MD06G1017400 and MD10G1255900) were detected in the QTL J06.1_sub1 and J10.2, respectively (Table S19). This protein is a component of SCF (ASK-cullin-F-box) E3 ubiquitin ligase complexes, which may mediate the ubiquitination and subsequent proteasomal degradation of target proteins (Zhu *et al.* 2017). Protein ubiquitination processes play an important role in plant–pathogen interactions (Lee *et al.* 2011). Re-sequencing and Sanger sequencing confirmed the SNPs and InDels in the *cis*-element and TF binding site of MD06G1017400 between the parents (Table S18). One SNP (KASP140) located in the TF binding site was significantly associated with the phenotype in the two pools (χ2= 5.718, *P* = 0.0168 <0.05) and 60 extreme accessions (χ2= 5.880, *P* = 0.0153 <0.05) (Table S20, TableS21, and Table S23). The expression of this gene in the fruit of hybrids from the resistant bulk 48 h after inoculation was consistently significantly higher than that of the susceptible bulk (Figure S15). While for another gene, MD10G1255900, a functional SNP (G-to-A) (KASP87) causing stop-gain was present in its CDS, and an enriched allele A was also present in the susceptible bulk and exhibited significant AFD between the two pools (χ2 = 5.203, *P* = 0.02255 <0.05) and the 60 extremes accessions (χ2 = 9.72, *P* = 0.001823 <0.05) (Table S19, Table S20, Table S21, and Table S23).

Ankyrin repeat-containing genes participate in the processes of signal transduction and protein kinase activity and thus may play an important role in the plant immune system (Mou *et al.* 2013; Ngaki *et al.* 2016). In the QTL of G11.1, an ankyrin repeat-containing gene (MD11G1116700) was identified in the peak (Table S19). The expression of this gene in the fruit of hybrids from the resistant bulk 48 h after inoculation was significantly higher than that of the susceptible bulk (Figure S15). The significant difference in expression was possibly caused by the validated mutants in the promoter region of this gene between parents (Figure S15 and Table S20). Furthermore, the KASP marker KASP126, developed in the candidate functional InDels, was significantly associated with the phenotype in the two pools (χ2= 5.966, *P* = 0.01459 < 0.05) and the 60 extreme accessions (χ2= 5.253, *P* = 0.02191 < 0.05) (Table S20, Table S21, and Table S23).

## Discussion

### The efficiency of the BAS-Seq strategy for QTL mapping in an outbreeding population

The double pseudo-testcross hypothesis was successfully applied to BSA mapping by separating the three types of markers. Data from either BSA-Seq or parental re-sequencing revealed three types of marker polymorphisms. The J- and G-type markers exhibit allelic heterozygosity and resemble the test cross for the maternal and paternal parent, respectively (Grattapaglia and Sederoff 1994). H-type markers exhibiting dual allelic heterozygosity are quite similar to the F_2_ progeny in inbreeding species (Ries *et al.* 2016; Takagi *et al.* 2013). The functional variations in the candidate genes controlling the target traits are also expected to possess the three types of polymorphisms (Bai *et al.* 2012b; Takos *et al.* 2006). Here, 13 major QTL were detected, four of which were from G, five of which were from J, and four of which were from the H polymorphism types (Table S14 and Figure 2-5). The predicted and validated functional mutations in the candidate genes also exhibited J-, G-, and H-type polymorphisms, which facilitates further gene exploration (Table S19 and Table S20). The QTL detected by using 3-type method exhibited the narrowest QTL intervals or the highest G’ value (File S2). Hence, BSA mapping by separating the three marker types allowed for the full utilization of polymorphic markers as well as the direct mapping of QTL on certain parent(s).

The GV method with a sliding window can realize noise reduction and unbiased statistical analysis for the three marker types. Three methods, namely SI, ED, and GV, are commonly used in BSA-Seq to reduce the noise and to measure the AFD between two extreme pools (Hill *et al.* 2013; Magwene *et al.* 2011; Takagi *et al.* 2013). In this study, 11 of the 12 significant QTL detected using the three methods coincided perfectly, indicating comparable effectiveness (Figure S5, Figure S6, Figure S7, and Table 2). However, the QTL profiles detected by GV were both narrower and more significant than those detected by SI (Table 2) and exhibited better unbiasedness and a lower false-positive ratio than that of ED (Table S11).

Sliding window analysis using different window sizes significantly narrowed down the QTL intervals. Fine-mapping based on large natural or pedigree segregation populations has been widely used but is both costly and labor-intensive/time-consuming (Bai *et al.* 2012a; Laurens *et al.* 2018; Terakami *et al.* 2016). It is very difficult to create recombinant inbred lines or near isogenic lines in outbreeding woody perennials, such as apple, pear, and peach, which greatly hinders fine mapping in these species (Di Pierro *et al.* 2016). Here we showed that a large amount of markers derived from the deeper re-sequencing of the two DNA pools made it possible to narrow down the QTL intervals with varied sliding window sizes analysis, *e.g.*, for resistance/susceptibility to isolate Zz26, the marker density is close to 1059/Mbp on average (Table 1 and Figure S1), which is almost 200 times more than conventional MapQTL methods (Gardner *et al.* 2014; Sun *et al.* 2015). By using varied sliding windows analysis, the spanning region of the QTL was narrowed down significantly from 1,639,342 bp to 707,246 bp, the averaged GV score increased from 5.55 to 6.03, and the stability of the QTL could also be evaluated (Figure S16 and Table S15).

Compared with the previous studies using the same segregating population, more QTL with much narrow interval were detected and a large number of previously reported QTL or major loci were reproduced in this study (Figure S11) (Cui *et al.*, 2014; Zhuang *et al.*, 2011). H14.1-Ls1, H14.1-Lw048 and H14.2-Zz26 coincided with Sf-g14-ls1, Sf-g14-lw048 (Table S14 and Figure S11) (Cui *et al.*, 2014). Sf-j10-zz26 was split into three narrower QTL, J10.1-Zz26, J10.2-Zz26 and J10.3-Zz26, showing that the accuracy on QTL identification is higher by BSA-seq than MapQTL based on low density linkage maps (Cui *et al.*, 2014). The pathogen isolate Mx1 was not used in this study because its weak pathogenicity to apple fruit (Zhuang *et al.*, 2011). Therefore, a few QTL or major loci reported by Cui *et al.* (2014) and Zhuang *et al.* (2011) were not presented.

### Multi-omics enables gene culling from candidates in QTL regions

Deep re-sequencing indicated a lot of genetic variation between the two parental cultivars, which is consistent with previous reports (Lee *et al.* 2016; Xing *et al.* 2016; Zhang *et al.* 2014). In the QTL intervals, genes lacking potential function-altering variations can be excluded. Transcriptomes can simultaneously indicate DEGs and genetic variations in CDS (Balan *et al.* 2018; Bonnet *et al.* 2017; Hill *et al.* 2013). In this study, 1,570 DEGs and 103,510 SNVs in the CDS of 24,533 genes were identified between the resistant and susceptible hybrids (Figure S14). These data, combined with large online transcriptome datasets, assisted the efficient excluding of many genes from the QTL intervals (Balan *et al.* 2017; Balan *et al.* 2018; Guo *et al.* 2017).

### The polygenetic signature of apple resistance to FRR provides evidence for the pathogenic variation among isolates

In this study, phenotyping was performed for five years, and hybrids with year-long robust resistance/susceptibility were selected for bulk creation. A total of 46 QTL, including 13 major QTL, were ultimately detected for resistance/susceptibility to the four *B. dothidea* isolates (Table S14). At least three major QTL for each pathogen isolate were found. These QTL demonstrated the apparent polygenetic signature of apple resistance to FRR, which corroborates previous reports (Cui *et al.* 2014; Zhuang *et al.* 2011). A variety of factors influence apple resistance to FRR, such as the size and density of the lenticels, the thickness of the wax layer, and the polyphenol content (Bai *et al.* 2015; Bénaouf and Parisi 2000; Fan *et al.* 2017; Li *et al.* 2013; Yu *et al.* 2014; Guan *et al.* 2015). Protein kinase plays an important role in signal transduction and pathogen recognition in the plant immune system (Park *et al.* 2012). Receptor-like protein kinases (RLPs) are widely reported to participate in plant–pathogen interactions (Kruijt *et al.* 2005). The NDR1 gene was once reported to confer resistance to *Pseudomonas syringae* pv. tomato DC3000 and plays an important role in defense against bacteria and viruses, and in the response to salicylic acid (Varet *et al.* 2003; Varet *et al.* 2002). MD06G1037600 (*MdNDR1*), MD10G1288500 (RLP), MD10G1288400 (Serine/threonine-protein kinase-like protein), and MD10G1169200 (Kinase-like protein TMKL), which are associated with the activation of disease resistance, pathogen recognition, and disease resistance signaling, were predicted as the most likely candidates determining resistance/susceptibility to FRR (Table 3 and Table S19).

Only four of the 46 QTL for resistance/susceptibility to the four isolates coincided or overlapped with others (Table S14,). The pathogenicity of *B. dothidea* differs significantly among isolates (Liu *et al.* 2011; Zhuang *et al.* 2011). The biological characteristics, pathogenicity, and internal transcribed spacer sequences differ significantly between pathogen strains of FRR (Lv *et al.* 2012). The diversity in resistance/susceptibility of apple to varied pathogen isolates supports the genetic differentiation of the pathogen according to the gene-for-gene hypothesis (Keen 1990; Van der Biezen and Jones 1998).

For a complex trait like apple ring rot disease resistance, large number of QTL each capture only a small proportion of the total genetic variance, and the pathogenicity varies dramatically among isolates (Cui *et al.*, 2014). The 46 QTL identified in this study can be developed into QTL based genomics-assisted breeding or can be used as diagnostic markers among high density SNP arrays, which enables empirical genomic selection (Bianco *et al.*, 2014; Bianco *et al.*, 2016). In addition, the candidate genes can help further research on the molecular mechanism of apple FRR resistance.
